# Morphometric Analysis of Human Embryonic Stem Cell-Derived Ventricular Cardiomyocytes: Determining the Maturation State of a Population by Quantifying Parameters in Individual Cells

**DOI:** 10.1155/2015/586908

**Published:** 2015-08-17

**Authors:** Harvey Y. S. Chan, Wendy Keung, Ronald A. Li, Andrew L. Miller, Sarah E. Webb

**Affiliations:** ^1^Division of Life Science and State Key Laboratory of Molecular Neuroscience, The Hong Kong University of Science and Technology, Clear Water Bay, Hong Kong; ^2^Stem Cell & Regenerative Medicine Consortium, Li Ka Shing Faculty of Medicine, The University of Hong Kong, Pokfulam, Hong Kong; ^3^Marine Biological Laboratory, Woods Hole, MA 02543, USA

## Abstract

Quantitative methods were established to determine the level of maturation of human embryonic stem cell-derived ventricular cardiomyocytes (hESC-vCMs) that were treated with different metabolic stimulants (i.e., isoproterenol and oleic acid) during early differentiation. Cells were double-immunolabeled with *α*-actinin and COX IV antibodies, to label the myofibrils and mitochondria, respectively, after which images were acquired via confocal microscopy. In order to determine the extent of differentiation, image analysis protocols were then used to quantify cell shape and area, as well as the degree of myofibrillar organization and intercalation of mitochondria between the myofibrils within the cells. We demonstrated that oleic acid or isoproterenol alone, or a combination of the two, induced a more elongated hESC-vCM phenotype than the untreated controls. In addition, cells treated with isoproterenol alone exhibited a similar level of myofibrillar organization as the controls, but those treated with oleic acid with/without isoproterenol exhibited a more organized (parallel) orientation of myofibrils. The combined isoproterenol/oleic acid treatment also resulted in enhanced intercalation of mitochondria between the myofibrils. We suggest that these quantitative morphometric methods might serve as simple and effective tools that can be utilized in the determination of the level of structural maturation of hESC-vCMs.

## 1. Introduction

In a recent study, data collected by the American Heart Association from 190 countries, confirmed that heart disease remains one of the main causes of premature death after stroke and cancer in many parts of the world today [1]. Terminal heart failure is caused by conditions such as ischemic heart disease, hypertension, and atrial fibrillation and is characterized by the irreversible loss of significant numbers of cardiomyocytes [2]. Furthermore, when cardiomyocytes malfunction due to disease (or aging), the limited regenerative nature of terminally differentiated cells in the adult human heart means that the repair of the myocardium is severely restricted, and this can be fatal for the patient. Many of the current treatments of heart disease are palliative in nature [3] and while heart transplantation is considered to be the last hope in many cases, it is severely limited by the lack of suitable donor organs [4]. Much of our current understanding of the mechanisms that regulate heart development in humans has been extrapolated from data acquired from animal model systems such as the mouse and zebrafish [5–9]. Even though these animal studies have yielded a lot of useful information regarding heart development, various species-specific differences have been identified [10]; thus, there is still much to be discovered specifically about the formation and regeneration of the human heart.

It has been less than two decades since embryonic stem cells (ESCs) were first successfully isolated from the inner cell mass of blastocyst-stage human embryos [11]. Thomson et al. [11] demonstrated that these cells have a normal karyotype and undergo undifferentiated proliferation but still maintain their pluripotent nature and are thus able to differentiate into endoderm, mesoderm, and ectoderm cells, depending on the external cues they receive. At this time, the significance of having readily available human embryonic stem cells (hESCs) for transplantation medicine, for drug discovery studies, and for basic developmental biology research was quickly appreciated. More recently, a method for reprogramming already differentiated cells (i.e., embryonic and adult fibroblasts) from mouse into pluripotent stem cells was described [12], shortly after which, two groups generated induced pluripotent stem cells (iPSCs) from human fibroblasts [13, 14]. Since then, the ready availability of hESCs and iPSCs as well as the effective differentiation of both these types of cells into cardiomyocytes [15–17] has provided scientists and clinicians with additional tools to study early heart development, as well as an* in vitro* model of cardiac disease. Furthermore, over the last few years, the idea of using cardiomyocytes in cell-replacement therapy has started to be considered for the treatment of heart disease [18, 19]. However, a lot of basic research is still required before this becomes a viable clinical option [20], largely because human ESCs and iPSCs appear to be structurally and functionally immature [15, 21–23]. For example, Kehat et al. [15] described the structural and functional properties of hESC-derived cardiomyocytes (hESC-CMs) as being reminiscent of early-stage cardiomyocytes, such that although several cardiac genes and transcription factors are expressed in these cells, as are various cardiac-specific proteins, the lack of organization of the myofibrils is a characteristic of the phenotype of immature cells. Rao et al. [22] also described the immature phenotype of both ESC- and iPSC-derived cardiomyocytes and attempted to induce a more mature phenotype in the latter, by growing cells on a polydimethylsiloxane (PDMS) scaffold containing microgrooves coated with fibronectin. They showed that although iPSC-derived cardiomyocytes did adopt a more mature phenotype when cultured under these conditions, the patterns of gene expression remained unchanged [22]. Most recently, Keung et al. [23] also characterized the functional and structural properties of hESC-derived ventricular cardiomyocytes (hESC-vCMs). They reported that whereas human adult cardiomyocytes are rod-shaped and in the order of 100 *μ*m in length, hESC-vCMs are relatively small in size and frequently round in shape (i.e., just 10–20 *μ*m in diameter). In addition, the rounded shape is maintained even during long periods in culture, although there is an increase in cell size over time. Keung et al. [23] also reported that the organization of contractile proteins in hESC-vCMs is poor such that myofibrils are at low density and organized in a random manner within the cytoplasm of these cells. It is thus clear from these various reports that it is especially important to characterize the morphological and ultrastructural properties of cells as they mature and in particular quantify parameters whenever possible, as this will help researchers to determine the differentiation status of cells and hence their ability to perform in cell replacement and transplantation procedures.

Even though the metabolic mechanisms that drive cardiac development are still largely unknown [24], a number of groups are starting to investigate the different biological cues that might be involved in driving maturation, which would allow strategies to expedite this process* in vitro* to be developed. In this study, hESC-vCMs were treated with isoproterenol, oleic acid, or a combination of both, and the effect of these pharmacological agents on cell maturation was compared with that of untreated (control) cells. Isoproterenol is a *β*-adrenoceptor agonist, which induces an influx of intracellular Ca^2+^ into cardiomyocytes and, in this way, stimulates cell contraction [15, 25–27], whereas oleic acid is a cis-monounsaturated long-chain fatty acid that is reported to play a cardioprotective role by modulating the lipid properties of the plasma membrane and by affecting cell signaling through its action on ion channel function [28]. Long-chain fatty acids such as oleic acid have also been shown to be an important source of energy that are required for cardiac function [29], as well as playing a role in signal transduction pathways in heart tissue [30] and promoting the maturation of energy metabolism in cardiomyocytes via activation of the peroxisome proliferator-activated receptor-*α* (PPAR-*α*) [31].

In our study, following treatment with isoproterenol and/or oleic acid, cells were immunolabeled with antibodies for *α*-actinin and COX IV to identify the myofibrils and mitochondria, respectively [32, 33], and then counterstained with DAPI to label the nuclei, and images were acquired via laser scanning confocal microscopy. Methods were then developed to determine the level of maturation of hESC-derived ventricular cardiomyocytes via quantification of four distinct morphological characteristics: cell shape, cell area, orientation of the myofibrils, and the level of intercalation of the mitochondria with the myofibrils.

## 2. Materials and Methods

### 2.1. Culture and Ventricular Specification of Human Embryonic Stem Cells

Ventricular cardiomyocytes were generated from the hES2 human embryonic stem cell (hESC) line as described previously [34]. In brief, undifferentiated hES2 cells were cultured alone (i.e., without feeder cells) in 6-well plates (Corning, Tewksbury, MA, USA), coated with Matrigel (Discovery Labware, Corning, New York, USA) and maintained in mTeSR1 medium (Stem Cell Technologies, Vancouver, BC, Canada). When the cells reached approximately 80% confluence, differentiation was initiated when hESC colonies were dissociated into single cells using Accutase (Invitrogen Life Technologies, Carlsbad, CA, USA) and then these were cultured in mTeSR1 medium with Matrigel (40 *μ*g/mL), BMP-4 (1 ng/mL, Invitrogen Life Technologies), and Rho kinase (ROCK) inhibitor (10 *μ*M; R&D Systems, Minneapolis, MN, USA) under hypoxic conditions for 24 h to form cardiogenic embryoid bodies (cardiospheres). The culture was then washed and incubated in StemPro34 SFM (Invitrogen Life Technologies) containing ascorbic acid (50 *μ*g/mL; Sigma-Aldrich Co. LLC., St. Louis, MO, USA), GlutaMAX-1 (2 mM; Invitrogen Life Technologies), BMP4 (10 ng/mL), and human recombinant activin-A (10 ng/mL; Invitrogen Life Technologies) for 3 days. On day 4, IWR-1 (4-(1,3,3a,4,7,7a-hexahydro-1,3-dioxo-4,7-methano-2H-isoindol-2-yl)-N-8-quinolinyl-benzamide), a small molecule Wnt inhibitor (5 *μ*M/mL; Enzo Life Sciences Inc., Farmingdale, NY, USA), was added to inhibit the canonical Wnt signaling pathway. On day 8, the culture was transferred to a normoxic environment and maintained in StemPro34 SFM containing 50 *μ*g/mL ascorbic acid alone.

At day 20 after the start of the cardiac differentiation process, cardiospheres were dissociated with Ca^2+^ and Mg^2+^-free PBS containing collagenase IV (1 mg/mL), DNase (10 *μ*g/mL), and 0.05% trypsin, after which they were transferred to Dulbecco's Modified Eagle Medium (DMEM, Invitrogen Life Technologies) containing 5% fetal calf serum (FCS), 2 mM glutamine, 1% penicillin/streptomycin, and 100 *μ*M nonessential amino acids (Invitrogen Life Technologies). The cultures were maintained for 24 h at 37°C in a humidified environment with 5% CO_2_/95% air. The cells were then transduced with the lentiviral construct LV-MLC2v-tdTomato-T2A-Zeo as described previously [35] and ventricular cardiomyocytes (hESC-vCMs) were specifically selected by incubation with zeocin (300 *μ*g/mL). The cells were allowed to recover from the zeocin selection procedure in DMEM containing normal 5% FCS for at least 96 h prior to the start of the metabolic treatment experiments.

### 2.2. Treatment of hESC-vCMs with Metabolic Stimulants

hESC-vCMs were plated onto glass cover slips (13 mm in diameter, No. 1.5H, Paul Marienfeld GmbH & Co. KG, Lauda-Königshofen, Germany) coated with poly-D-lysine (5 *μ*g/cm^3^) and Matrigel at a density of 4 × 10^4^ cells per cover slip. After 24 h, the cells were treated with 0.3 *μ*M isoproterenol (Sigma-Aldrich Co. LLC.) and/or either 100 *μ*M or 200 *μ*M oleic acid (Sigma-Aldrich Co. LLC.) for 96 h before they were fixed with 4% paraformaldehyde in phosphate-buffered solution (PBS; 137 mM NaCl, 2.68 mM KCl, 16 mM Na_2_HPO_4_, 5.2 mM NaH_2_PO_4_, pH 7.3) for 15 min. The fixed cells were then washed thoroughly with PBS and then stored in PBS at 4°C prior to immunolabeling.

### 2.3. Immunolabeling

All the incubation steps used in this protocol were performed in the dark at room temperature (i.e., ~22°C). The isoproterenol and/or oleic acid-treated and paraformaldehyde-fixed hESC-vCMs were incubated in PBS containing 0.1% Triton X-100 (PBS-T) for 10 min, after which they were incubated in blocking buffer (PBS-T containing 10% goat serum and 1% bovine serum albumin; BSA) for 30 min. The cells were then incubated sequentially with the mouse monoclonal anti-sarcomeric *α*-actinin antibody (at a dilution of 1 : 100; Abcam, Cambridge, UK) and the rabbit polyclonal anti-COX IV antibody (at a dilution of 1 : 800; Abcam). Both antibodies were diluted just prior to use with blocking buffer. Alpha-actinin was then visualized using the Alexa Fluor 488 F(ab′)_2_ fragment of the goat anti-mouse IgG (H + L) antibody (at a dilution of 1 : 200; Invitrogen Life Technologies). COX IV was visualized using an ATTO 647N (STED/GSD) goat anti-rabbit IgG antibody (at a dilution of 1 : 200; Active Motif, Carlsbad, CA, USA). Each antibody incubation step lasted for 1 h; between each incubation the hESC-vCMs were rinsed extensively with wash buffer (PBS-T containing 1% goat serum and 0.1% BSA). Following a final rinse with wash buffer, the cells were rinsed once with Milli-Q water and then they were mounted under ProLong Gold antifade reagent containing DAPI (Invitrogen Life Technologies). The mounted cells were kept in the dark overnight at room temperature and subsequently transferred to 4°C before visualization via confocal laser scanning and multiphoton excitation microscopy.

### 2.4. Confocal Laser Scanning and Multiphoton Excitation Microscopy

The fluorescence images of labeled cardiomyocytes were acquired with a Leica TCS SP5 II laser scanning confocal system mounted on a Leica DMI 6000 inverted microscope (Leica Microsystems, Wetzlar, Germany) equipped with multiphoton excitation capacity. Images were obtained using a Leica HCX PL APO 40x/1.25–0.75 NA oil-immersion objective lens. Alexa Fluor 488 fluorescence was observed via confocal microscopy using 488 nm excitation and 500–550 nm emission, tdTomato fluorescence was observed via confocal microscopy using 561 nm excitation and 575–610 nm emission wavelengths, and ATTO 647N fluorescence was observed via confocal microscopy using 633 nm excitation and 650–750 nm emission. DAPI was visualized via multiphoton excitation microscopy using 780 nm excitation and 432–482 nm emission.

### 2.5. Data Analysis

Following immunolabeling and confocal microscopy, the images of the cardiomyocytes generated were analyzed using Image J (National Institutes of Health, Bethesda, MD, USA). The shape of cardiomyocytes was quantified using the “circularity” function. Images of cells showing *α*-actinin labeling were used for this analysis. The confocal microscope-generated scale bar included in each image was used to calibrate the scale in Image J. The perimeter of the cell was then traced using the “Freehand Selections” tool and the shape of the cell was subsequently determined, such that a more rounded cell exhibited a circularity value close to 1 whereas a more elongated cell had a value nearer to 0. Quantitative data analyses were carried out using Microsoft Office Excel 2010 (Microsoft, Redmond, WA, USA).

To quantify the orientation of the myofibrils in cardiomyocytes, the “Multipoint” tool in Image J was used to analyze the *α*-actinin labeled images. In each cell, a total of 32 *x*, *y* coordinates was applied to mark two points along the length of each of 16 myofibrils. These coordinates were exported to Excel and the slope (m) of the line connecting the two points was calculated using the following equation: *m* = (*y*_1_ − *y*_2_)/(*x*_1_ − *x*_2_). The variance (i.e., standard deviation^2^) of these calculated slope values was then used to indicate if myofibrils were running in parallel through a cell (in which case, they would have a variance near to zero) or if they were distributed in a random manner (such that they would exhibit a relatively higher variance value).

To determine the level of intercalation between the *α*-actinin-labeled myofibrils and the COX IV-labeled mitochondria, a line scan analysis was performed. The individual confocal fluorescent images of *α*-actinin and COX IV were opened in Image J and then stacked. The microscope-generated scale bar was used to set the scale, and four straight lines of between 20 *μ*m and 30 *μ*m in length and ~1 *μ*m in width were placed in areas of each cardiomyocyte where the myofibrils were arranged in parallel such that the lines were perpendicular to the orientation of the myofibrils. In addition, lines were placed away from both the nucleus and the outer edge of the cell. Two sets of numerical line scan data were acquired for each line: one for the *α*-actinin image and the other for the COX IV image. These data were then exported to Excel for graph plotting and calculation of the Pearson correlation coefficient.

Dunnett's test was used to determine statistically significant differences between the data sets. This was performed with the SPSS statistics software (IBM, Armonk, NY, USA).

## 3. Results

The effect of isoproterenol and oleic acid on hESC-vCM maturation was determined by quantifying the cell shape, cell area, orientation of the myofibrils, and the level of intercalation of the mitochondria with the myofibrils. In these experiments, a total of 309 cells was analyzed from 4 different experiments.

### 3.1. Quantification of Cell Shape

**Figure 1:**
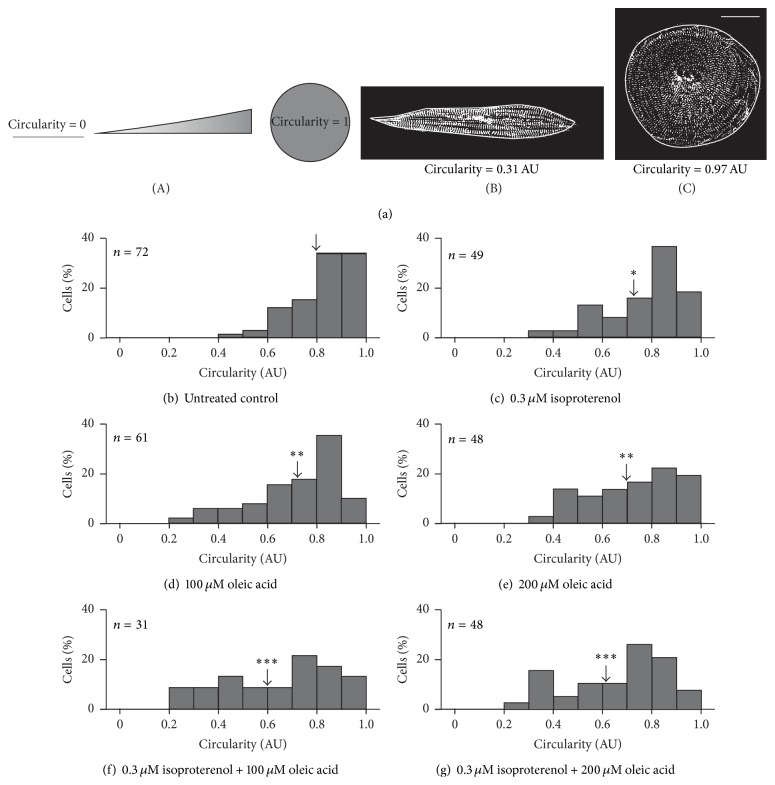
Effect of isoproterenol and oleic acid on the shape of hESC-vCMs. ((a) panel (A)) Schematic illustration to show that circularity values of 0 and 1 represent a straight line and perfect circle, respectively. ((a) panel (B), (a) panel (C)) Representative examples of elongated and rounded hESC-vCMs (labeled with an *α*-actinin antibody) that have circularity values of ((a) panel (B)) 0.31 AU and ((a) panel (C)) 0.97 AU, respectively. Scale bar is 30 *μ*m. (b–g) Histograms to show the distribution of cells (in %) exhibiting a particular circularity value in (b) untreated controls and (c–g) following treatment with (c) 0.3 *μ*M isoproterenol, (d) 100 *μ*M oleic acid, (e) 200 *μ*M oleic acid, (f) 0.3 *μ*M isoproterenol plus 100 *μ*M oleic acid, or (g) 0.3 *μ*M isoproterenol plus 200 *μ*M oleic acid. The number of cells analyzed is indicated in the upper left corner of each graph. The arrows indicate the mean circularity value determined for the population of cells, and the asterisks indicate values that are significantly different from the untreated control at ^*∗*^ *p* ≤ 0.05; ^*∗∗*^ *p* ≤ 0.01; and ^*∗∗∗*^ *p* ≤ 0.001.

hESC-vCMs were either untreated or were treated with 0.3 *μ*M isoproterenol; 100 *μ*M or 200 *μ*M oleic acid; or 0.3 *μ*M isoproterenol and either 100 *μ*M or 200 *μ*M oleic acid. The cells were then immunolabeled with an *α*-actinin antibody to identify the myofibrils, after which the shape of the cells was quantified by determining the circularity value such that a value of 1 indicates a perfect circle whereas a value of 0 indicates a straight line (Figure 1(a) panel (A)). Representative examples of cells treated with 0.3 *μ*M isoproterenol and 200 *μ*M oleic acid, which induces the greatest variation in shape of all the treatments, are shown in Figures 1(a) panel (B)-1(a) panel (C). These cells have circularity values of (Figure 1(a) panel (B)) ~0.31 Arb. Units (AU) and (Figure 1(a) panel (C)) ~0.97 AU, respectively, with the cell in Figure 1(a) panel (B) showing a relatively elongated morphology, while that in Figure 1(a) panel (C) is more rounded.

A range of circularity values was observed whether the hESC-vCMs were untreated or treated with isoproterenol and/or oleic acid; these are shown in the series of histograms in Figures 1(b)–1(g).  Figure 1(b) shows the range of circularity values obtained for the untreated control cells (*n* = 72), such that ~64.0% of cells exhibited circularity values of between 0.8 and 1.0 Arb units (AU); ~30.5% of cells exhibited circularity values between 0.6 and 0.8 AU, and ~5.5% of cells exhibited circularity values between 0.4 and 0.6 AU. The mean circularity was calculated to be 0.8 AU (see black arrow in Figure 1(b)). When cells were treated with 0.3 *μ*M isoproterenol (*n* = 49; Figure 1(c)), however, there was an overall shift in the shape of cells from a more rounded to a more elongated phenotype, when compared with the untreated controls. This is reflected in a lower mean circularity value of 0.73 AU (see black arrow in Figure 1(c)). When treated with 0.3 *μ*M isoproterenol, ~43.0% of the cells exhibited circularity values of between 0.8 and 1.0 AU, ~32.6% of cells were between 0.6 and 0.8 AU, ~18.4% of cells were between 0.4 and 0.6 AU, and ~6.0% of cells exhibited circularity values between 0.3 and 0.4 AU (Figure 1(c)). Indeed, the circularity values of cells treated with 0.3 *μ*M isoproterenol were significantly lower than those of the untreated control at *p* ≤ 0.05 using Dunnett's test. Figures 1(d) and 1(e) show the spread of circularity values in cells treated with 100 *μ*M and 200 *μ*M oleic acid, respectively, and the black arrows in these figures indicate mean values of 0.72 AU and 0.70 AU, respectively. When cells were treated with 100 *μ*M oleic acid (*n* = 61; Figure 1(d)), the proportion exhibiting circularity values between 0.8 and 1.0 AU, 0.6 and 0.8 AU, 0.4 and 0.6 AU, and 0.2 and 0.4 AU was ~41.0%, ~36.0%, ~16.4%, and ~6.6% of cells, respectively. In addition, following treatment with 200 *μ*M oleic acid (*n* = 48; Figure 1(e)), the proportion of cells exhibiting circularity values between 0.8 and 1.0 AU, 0.6 and 0.8 AU, 0.4 and 0.6 AU, and 0.2 and 0.4 AU was ~35.4%, ~35.4%, ~25.0%, and ~4.2%, respectively. Thus, the 100 *μ*M and 200 *μ*M oleic acid treatments both generated significantly more cells in the population with a lower circularity value, than those in the untreated control population at *p* ≤ 0.01 using Dunnett's test. Figures 1(f) and 1(g) show the spread of circularity values in cells treated with 0.3 *μ*M isoproterenol and oleic acid at either 100 *μ*M (*n* = 31; Figure 1(f)) or 200 *μ*M (*n* = 48; Figure 1(g)). With 0.3 *μ*M isoproterenol and 100 *μ*M oleic acid (Figure 1(f)), the proportion of cells exhibiting circularity values between 0.8 and 1.0 AU, 0.6 and 0.8 AU, 0.4 and 0.6 AU, and 0.2 and 0.4 AU was ~22.6%, ~22.6%, ~25.8%, and ~29.0%, respectively, and a mean circularity value of 0.6 AU was calculated (see black arrow in Figure 1(f)). With 0.3 *μ*M isoproterenol and 200 *μ*M oleic acid (Figure 1(g)), the proportion of cells exhibiting circularity values between 0.8 and 1.0 AU, 0.6 and 0.8 AU, 0.4 and 0.6 AU, and 0.2 and 0.4 AU was ~23.0%, ~37.5%, ~18.7%, and ~20.8%, respectively, and a mean circularity value of 0.62 AU was calculated (see black arrow in Figure 1(g)). Thus, both of the 0.3 *μ*M isoproterenol/100 *μ*M oleic acid and 0.3 *μ*M isoproterenol/200 *μ*M oleic acid combined treatments generated significantly more cells in the population with a lower circularity value than those in the untreated control population at *p* ≤ 0.001.

### 3.2. Quantification of Cell Area

**Figure 2:**
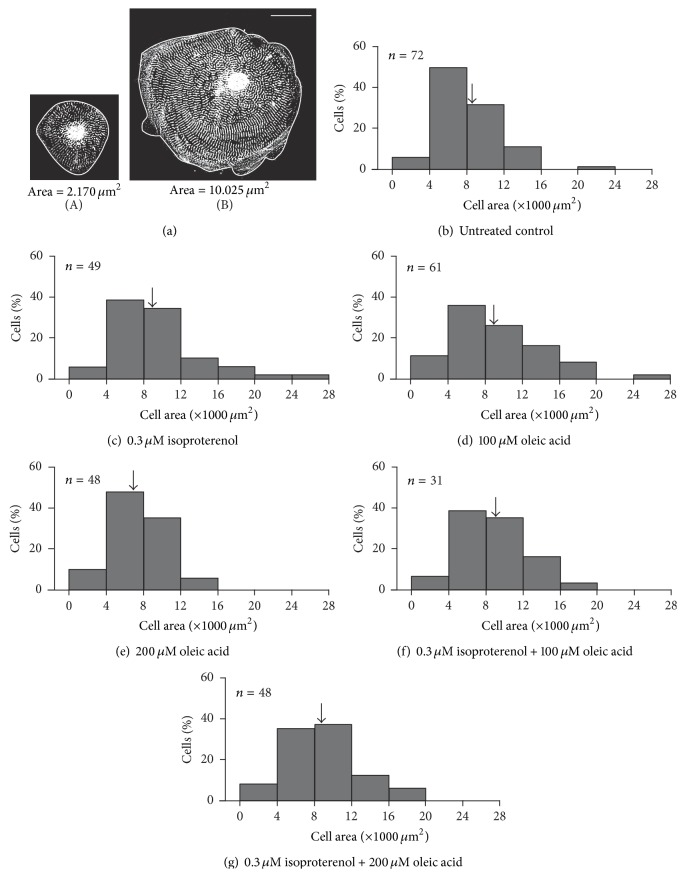
Effect of isoproterenol and oleic acid on the area of hESC-vCMs. ((a) panel (A), (a) panel (B)) Representative examples of the typical areas of hESC-vCMs measured. These two cells (labeled with an *α*-actinin antibody) have cell areas of ((a) panel (A)) 2,170 *μ*m^2^ and ((a) panel (B)) 10,025 *μ*m^2^, respectively. Scale bar is 30 *μ*m. (b–g) Histograms to show the distribution of cells (in %) exhibiting a particular cell area in (b) untreated controls and (c–g) following treatment with (c) 0.3 *μ*M isoproterenol, (d) 100 *μ*M oleic acid, (e) 200 *μ*M oleic acid, (f) 0.3 *μ*M isoproterenol plus 100 *μ*M oleic acid, or (g) 0.3 *μ*M isoproterenol plus 200 *μ*M oleic acid. The number of cells analyzed is indicated in the upper left corner of each graph. In all treatment groups, none of the area values was significantly different from those of the untreated controls. The arrows indicate the mean cell area determined for the population of cells.

The effect of isoproterenol and/or oleic acid treatment on cell area was also investigated using the same populations of cells used for the cell shape measurements. Similar to the circularity data, a range of cell area values was obtained for each treatment as well as for the untreated controls. The two representative untreated hESC-vCMs shown in Figure 2 have areas of ~2,170 *μ*m^2^ (Figure 2(a) panel (A)) and ~10,025 *μ*m^2^ (Figure 2(a) panel (B)).

The range of cell areas measured for each treatment is shown in the series of histograms in Figures 2(b)–2(g).  Figure 2(b) shows the range of areas measured in the untreated control cells, such that ~5.5% of the cells exhibited areas less than 4,000 *μ*m^2^, ~50.0% exhibited areas between 4,000 *μ*m^2^ and 8,000 *μ*m^2^, ~32.0% had areas between 8,000 *μ*m^2^ and 12,000 *μ*m^2^, ~11.1% had areas between 12,000 *μ*m^2^ and 16,000 *μ*m^2^, and ~1.4% of cells exhibited areas between 20,000 *μ*m^2^ and 24,000 *μ*m^2^. A mean cell area of 8,109 *μ*m^2^ was calculated (see black arrow in Figure 2(b)). Figures 2(c)–2(g) show the effects of the various treatments on the cell area. In all cases, a similar range of areas was calculated as for the untreated controls. For example, in all cases, between 6% and 11% of cells had an area less than 4,000 *μ*m^2^; the majority of cells (i.e., 62% to 83%, depending on the treatment) had areas of 4,000 *μ*m^2^ and 12,000 *μ*m^2^ such that 35% to 48% had areas between 4,000 *μ*m^2^ and 8,000 *μ*m^2^ and 26% to 37.5% had areas between 8,000 *μ*m^2^ and 12,000 *μ*m^2^_._ Of the remaining cells, 6.25% to 16% had areas between 12,000 *μ*m^2^ and 16,000 *μ*m^2^, 3% to 8% had areas between 16,000 *μ*m^2^ and 20,000 *μ*m^2^, and 1.5% to 4% of cells had areas between 20,000 *μ*m^2^ and 28,000 *μ*m^2^. In addition, mean cell areas of 9,358 *μ*m^2^, 9,014 *μ*m^2^, 7,383 *μ*m^2^, 9,007 *μ*m^2^, and 8,884 *μ*m^2^ were calculated for cells treated with 0.3 *μ*M isoproterenol, 100 *μ*M oleic acid, 200 *μ*M oleic acid, 0.3 *μ*M isoproterenol plus 100 *μ*M oleic acid, and 0.3 *μ*M isoproterenol plus 200 *μ*M oleic acid, respectively (see black arrowheads in Figures 2(c)–2(g)). None of the treatments yielded area results that were significantly different from the untreated controls using Dunnett's test.

### 3.3. Quantifying the Orientation of the Myofibrils

**Figure 3:**
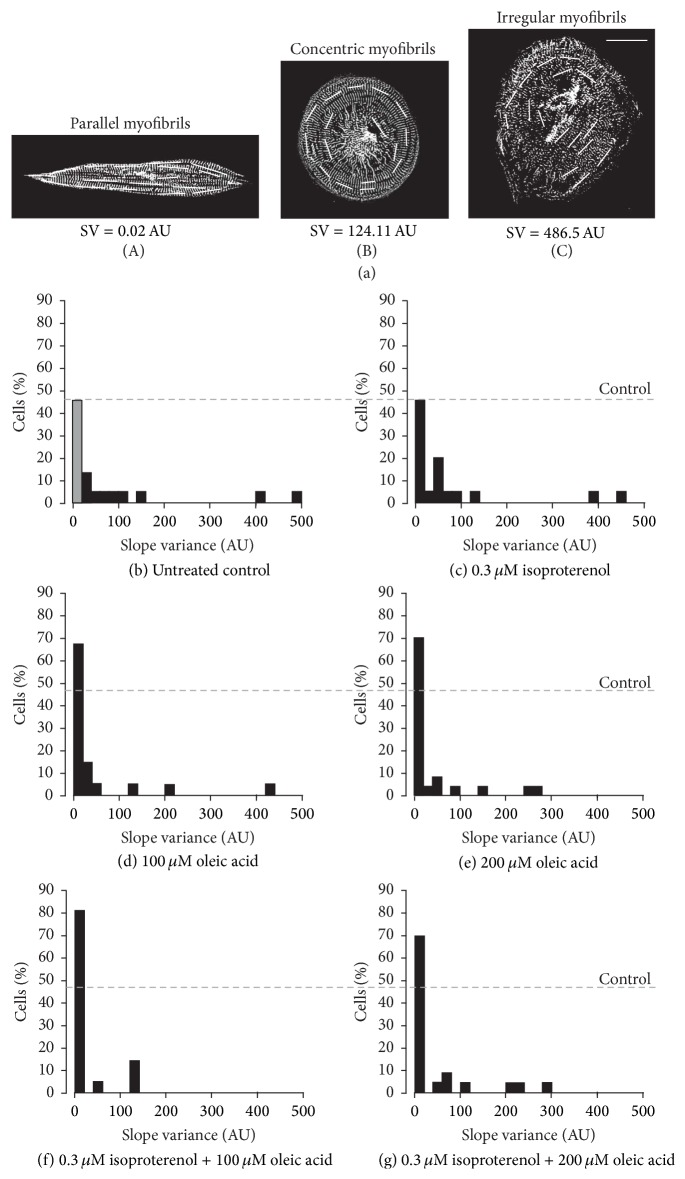
Effect of isoproterenol and oleic acid on the orientation of myofibrils in the hESC-vCMs. ((a) panel (A)–(a) panel (C)) The orientation of the myofibrils localized within hESC-vCMs was determined by calculating the slope of lines placed along the length of the myofibrils. ((a) panel (A)) hESC-vCMs with the myofibrils arranged in parallel exhibited a low slope variance (SV) value (e.g., 0.02 AU), when compared with ((a) panel (B)) cells where the myofibrils were arranged in a concentric manner and exhibit an SV of ~124 AU or ((a) panel (C)) where the myofibrils were arranged in an irregular orientation when the SV was greater again (e.g., ~480 AU). Scale bar is 30 *μ*m. (b–g) Histograms to show the distribution of cells (in %) exhibiting a particular variance value in (b) untreated controls and (c–g) following treatment with (c) 0.3 *μ*M isoproterenol, (d) 100 *μ*M oleic acid, (e) 200 *μ*M oleic acid, (f) 0.3 *μ*M isoproterenol plus 100 *μ*M oleic acid, or (g) 0.3 *μ*M isoproterenol plus 200 *μ*M oleic acid. *N* = 20 for each treatment group and the untreated controls. The grey bar and grey dashed lines indicate the percentage of cells in the untreated control group with the lowest myofibrillar SV value.

The effect of isoproterenol and/or oleic acid on the organization of myofibrils was also investigated (Figure 3). Using the *α*-actinin images used for the cell shape and area measurements, a series of short lines was drawn on top of and in the same orientation as the myofibrils (i.e., perpendicular to the sarcomeric *z*-lines) in each cell (Figures 3(a) panel (A)–3(a) panel (C)). The value of the slope of each line was then calculated, after which the variance of the slopes was determined. Cells with randomly oriented myofibrils exhibit a relatively high variance value whereas those where myofibrils are arranged in parallel have variance values approaching zero. Figures 3(a) panel (A)–3(a) panel (C) show three representative examples of untreated control hESC-vCMs to illustrate the different patterns of myofibrillar organization; these cells have slope variances of ~0.02 AU (Figure 3(a) panel (A)), ~124 AU (Figure 3(a) panel (B)), and ~486 AU (Figure 3(a) panel (C)).

The range of slope variances measured for each treatment and the untreated controls is shown in the series of histograms in Figures 3(b)–3(g). Whereas a relatively wide range of variance values was observed for the untreated control and each treatment group, we wanted to determine if any of the treatments stimulated the formation of more parallel myofibrils, when compared with the untreated control group. Thus, we were most interested in the percentage of cells with slope variance values between 0 AU and 20 AU. In the untreated controls (*n* = 22), ~46% cells had a variance value of 0–20 AU (Figure 3(b)). Similarly, when cells were treated with isoproterenol alone (*n* = 19), ~47% had a variance value from 0 to 20 AU (Figure 3(c)). When cells were treated with 100 *μ*M oleic acid (*n* = 21; Figure 3(d)) or 200 *μ*M oleic acid (*n* = 23; Figure 3(e)), however, ~67% and ~70% cells, respectively, had variance values between 0 AU and 20 AU. Furthermore, when cells were treated with isoproterenol and 100 *μ*M oleic acid (*n* = 21; Figure 3(f)) or 200 *μ*M oleic acid (*n* = 23; Figure 3(g)), ~81% and ~70% cells, respectively, had variance values between 0 AU and 20 AU. Thus, treatment with oleic acid appeared to stimulate a larger number of cells in the population to possess myofibrils that were oriented in parallel.

### 3.4. Quantifying the Level of Intercalation of the Mitochondria with the Myofibrils

**Figure 4:**
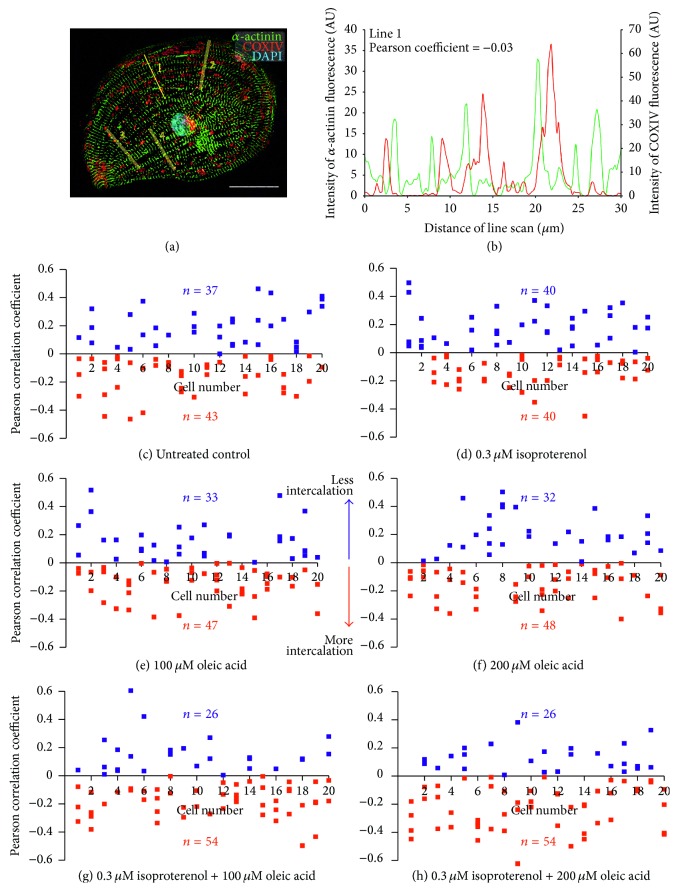
Effect of isoproterenol and oleic acid on the intercalation of the mitochondria between the myofibrils. (a) Representative example of an hESC-vCM that had been immunolabeled with antibodies to *α*-actinin and COX IV and costained with DAPI, to label the myofibrils, mitochondria, and nucleus, respectively. Four line scan analyses were then performed per cell, such that the lines were placed perpendicular to the orientation of the myofibrils and were kept away from the nucleus. Scale bar is 30 *μ*m. (b) Representative line graph to show the intensity of fluorescence of *α*-actinin (green) and COX IV (red) labeling along line 1 from panel A. The Pearson correlation coefficient (PCC) was calculated to be −0.03. (c–h) Series of scatter graphs to show the PCC determined for each of 4 lines in *n* = 20 cells in (c) untreated controls and (d–h) following treatment with (d) 0.3 *μ*M isoproterenol, (e) 100 *μ*M oleic acid, (f) 200 *μ*M oleic acid, (g) 0.3 *μ*M isoproterenol plus 100 *μ*M oleic acid, or (h) 0.3 *μ*M isoproterenol plus 200 *μ*M oleic acid. Numbers in blue and red indicate line scans exhibiting positive and negative PCC values, respectively, the latter indicating a higher level of intercalation between the COX IV labeled mitochondria and the *α*-actinin-labeled myofibrils.

Figure 4(a) shows a representative example of an hESC-vCM that was immunolabeled with antibodies to *α*-actinin and COX IV to label the myofibrils and mitochondria, respectively. The cell was costained with DAPI to label the nucleus. Line 1 indicates the location of one of four line scan analyses that were conducted on this cell to determine the level of intercalation of the mitochondria with the myofibrils. In every cell that was tested for both the treatments and the untreated controls, four lines were placed away from the nucleus and perpendicular to the orientation of the myofibrils. Figure 4(b) shows the two line graphs produced from the line scan analysis performed along line 1 in Figure 4(a), such that the green and red lines show the fluorescence intensity of *α*-actinin and COX IV, respectively. The level of intercalation was then determined by calculating the Pearson correlation coefficient (PCC), which in this case equals ~−0.03. The PCC ranges from +1 to −1, where +1 indicates a positive correlation and −1 indicates a negative correlation or perfect exclusion, with zero indicating no correlation (i.e., random distribution). Since the images have a black background, the PCC will never be exactly +1 or −1; however, a higher level of intercalation of mitochondria with myofibrils is indicated by a more negative PCC value.

Figures 4(c)–4(h) are a series of scatter graphs showing the PCC data from 80 line scans (4 line scans conducted per cell in 20 cells) in the untreated controls and in each of the 5 different treatment groups. In the untreated (control) group, 37 line scans exhibited a positive PCC, whereas 43 line scans had a negative PCC (Figure 4(c)). Similarly, in the 0.3 *μ*M isoproterenol group, there was an exact 1 : 1 split between positive and negative PCC values such that 40 lines exhibited positive PCC values and 40 had negative values (Figure 4(d)). In the 100 *μ*M and 200 *μ*M oleic acid treatment groups, the PCC values obtained were almost identical such that 33 and 32 lines exhibited positive PCC values, respectively, and 47 and 48 lines exhibited negative values, respectively (Figures 4(e) and 4(f)). In addition, in the 0.3 *μ*M isoproterenol and 100 *μ*M oleic acid, and the 0.3 *μ*M isoproterenol and 200 *μ*M oleic acid groups, the number of line scans with positive and negative PCC values was identical, such that, in both cases, 26 lines had positive PCC values whereas 54 lines had negative values (Figures 4(g) and 4(h)).

## 4. Discussion

It has been previously reported by several groups that embryonic stem cells induced to differentiate into cardiac lineage cells undergo a change in size and shape as they mature [5, 23, 36–38]. Differentiation is also accompanied by changes in the ultrastructure, cell cycle, and metabolic properties of the cell. In the developing mouse heart, for example, it has been reported that early embryonic cardiomyocytes are polygonal in shape and the myofibrils are oriented randomly throughout the cytoplasm; however, during fetal development, these cells become more elongated and the myofibrils become progressively more aligned, ultimately developing a very well organized cytoarchitecture [5]. More recently, Lundy et al. [38] demonstrated that late-stage human ESC-CMs and iPSC-CMs (i.e., at between 80 and 100 days of* in vitro* differentiation and culturing) are larger in size and exhibit a greater level of anisotropy, as well as a higher density of myofibrils and a more organized pattern of sarcomeres, when compared with early-stage cells of 20–40 days. In addition, cardiomyocytes isolated from mature tissues and placed in culture conditions undergo specific morphological changes that are associated with dedifferentiation. For example, when human adult atrial and ventricular myocytes are isolated from patients undergoing cardiac surgery, the cells are initially rod shaped [39]. However, on dedifferentiation, the cells undergo a loss of sarcomeric structure and concomitant change in shape. When these cells are subsequently cultured in low serum, they can then redifferentiate such that all the cells flatten and spread and they either become large in size, with nascent myofibrils, or smaller with a more mature pattern of myofibrillar organization [39].

Before cells can be used for clinical purposes, their phenotypic properties need to be characterized in detail. Indeed, it has commonly been agreed that the morphological and ultrastructural properties of cells must be investigated as these are key characteristics that determine the differentiation status of cells and hence their ability to perform in cell replacement and transplantation procedures [36]. The importance of making precise observations and thus acquiring detailed information regarding the changes that occur during differentiation at the level of individual cells has been emphasized previously [40]. Indeed, in recent years, confocal laser scanning microscopy has become a popular tool for exploring different aspects of cardiomyocyte differentiation. These include studies of the migration of neural crest-derived stem cells in the developing heart field and their subsequent differentiation into cardiomyocytes [41], and the successful transplantation of bone marrow-derived cardiomyocytes expressing myosin light chain-2v-derived GFP into the mouse heart [42]. Such observations have helped to elucidate, at a cellular level, the mechanisms and pathways that are involved in the formation of a functional heart during development [5]. It is also crucial, however, to confirm single cell observations that might be more qualitative in nature, with quantitative information regarding the precise nature of maturity of cells. Indeed, here we immunolabeled hESC-vCMs, used confocal microscopy to acquire images of the cells, and then developed quantitative methods to determine their level of maturity. In these experiments, all the cells were of a similar age (i.e., approximately 20 to 30 days; what Lundy et al. [38] classed as an “early” stage of differentiation) and we tested the effect of two metabolic agents (i.e., isoproterenol and oleic acid) on the maturity of cardiomyocytes, when compared with untreated (control) cells. As it would be impractical to acquire high-magnification confocal images from the large numbers of cells that are required for other types of experiments, such as some biochemical assays, our n-numbers are not large. In addition, it is clear that for all the parameters we measured, a range of effects was observed within the population and it is for this reason that we presented the results in either histogram or scatter diagram form. It was therefore important to show the data in this way to emphasize the fact that the individual cells within a population do show morphological variation and thus simply presenting mean values does not necessarily give an accurate measure of cellular phenotype.

Cell shape was the first parameter that we measured to analyze the maturity of cardiomyocytes (Figure 1). A so-called “shape factor” (what we call circularity value) has previously been utilized when comparing the shape of H7- and RuES-2-derived hESC-CMs during early (i.e., 20–40 days) and late (i.e., 80–120 days) differentiation [38]. The shape factor has also been used to determine the level of differentiation of hESCs but in this case it was the shape of the nuclei that was quantified [43]. Our data showed that in the untreated control group, the majority of cells had a more rounded morphology, with around 70% of the population exhibiting a circularity of 0.8 arbitrary units (AU) and above. In all the treatment groups (i.e., the isoproterenol or oleic acid alone groups, or the isoproterenol plus oleic acid groups), a subset of cells also had a circular phenotype. However, in each treatment group a higher proportion of cells had a more elongated morphology, indicating a more mature phenotype. Typically, adult ventricular cardiomyocytes from human biopsies are rod-shaped [39] and so, using images of such cells published previously [44, 45], we determined the circularity value to be ~0.5 AU. In our measurements, however, the lowest circularity values we measured were in the range of 0.2–0.3 AU and the cells exhibited a more spindle-shaped morphology (Figure 1(a) panel (B)). As the shape of cardiomyocytes is proposed to have a direct impact on their contractility, via the way that the sarcomeres are registered laterally [37], we suggest that the difference in circularity values obtained for adult cardiomyocytes and the maturing cardiomyocytes used in our study might reflect the differences in maturity of these cells. When comparing the various treatment groups, oleic acid alone had a more profound effect on cell shape than isoproterenol but a combination of the two together stimulated a higher proportion of the cells to exhibit a more elongated morphology than when either was used alone. Our data suggest that providing a source of energy to the cells and promoting the maturation of energy metabolism (via oleic acid [29, 31]) might be more important at these early stages of maturation than stimulating an influx of Ca^2+^ (via isoproterenol [25, 26]) but that when both are used together, their different actions are complementary to help promote cell elongation and thus maturation.

Although both isoproterenol and oleic acid clearly affected the cell shape, neither of them (either when used alone or together) appeared to have a significant effect on the cell area (Figure 2). Similar to the cell shape data, a range of different cell areas was observed for the controls and in each treatment group such that the smallest and largest cell areas were ~2,000 *μ*m^2^ and ~24,000–28,000 *μ*m^2^, respectively. However, the spread of area values was very similar in all the groups, indicating that not one treatment affected the cell area any more or less than the others. Previously, Lundy et al. [38] reported that the size of H7- and RuES-2-derived hESC-CMs increased from ~480 *μ*m^2^ to ~1,700 *μ*m^2^ from early (20–40 day) to late (20–40 day) stages, respectively, using similar methods to those we employed. The size of the hESC-vCMs derived from hES2 cells that we were using appeared to be at least 10–20-fold larger than those described previously [38] (i.e., the majority of the untreated control hES2-derived ventricular cardiomyocytes were ~4,000–8,000 *μ*m^2^ when compared with just ~480 *μ*m^2^ of H7 and RuES-2-derived cardiomyocytes, at a comparable stage of differentiation). Again using images published previously [44, 45], we determined that human adult ventricular cardiomyocytes have a typical cell area of ~4,300 *μ*m^2^, which is somewhat similar to the area values that we obtained. We suggest that the cell area differences observed between our data and those previously reported might be due to the different source of stem cells and/or the fact that the concentration of cells plated out at the start of the experiment varied [38].

It has been previously reported that the alignment of myofibrils in immature cardiomyocytes is random and relatively disorganized, whereas mature cardiomyocytes exhibit a well-aligned myofibrillar cytoarchitecture [24, 36, 38, 46–49]. These previous studies, however, did not quantify the level of organization of myofibrils in the cells. In our data analysis, on the other hand, we quantified the slope variance of myofibrils in the untreated control cell group as well as in those treated with isoproterenol or oleic acid alone, or with a combination of the two (Figure 3). Cells with randomly oriented myofibrils were expected to exhibit a relatively high variance value whereas those where the myofibrils were arranged in parallel were expected to have variance values approaching zero. We were thus most interested in determining the percent of cells with the lowest variance values (i.e., between 0 and 20 AU; see grey bar in Figure 3(b)). A similar method was previously used to determine the alignment of human iPSC-CMs on microgrooved and non-microgrooved scaffolds, whereby the variance values of the angle of the long axis of the nuclei relative to the horizontal axis of the image field were measured and the smaller the value then the more well-aligned the population of cells was determined to be [22]. In our experiments, we found that the percent of isoproterenol-treated cells that exhibited more parallel myofibrils was very similar (i.e., ~46%) to that of the untreated controls (i.e., ~47%), suggesting that this metabolic stimulant does not have any significant effect on the development of myofibrillar organization in these cells. On the other hand, when cells were treated with oleic acid, either alone (at 100 *μ*M or 200 *μ*M) or in combination with isoproterenol, a higher percentage of cells exhibit more parallel myofibrils (i.e., from ~68% to ~80%, depending on the treatment; Figures 3(d)–3(g)). These results therefore indicate that oleic acid might stimulate this key aspect of cardiomyocyte maturation.

Cardiogenesis, whether it occurs during embryogenesis or during heart repair, is known to involve the differentiation of noncontractile stem cells with few energy requirements, into energetically proficient contracting cardiomyocytes [24]. This transition therefore requires the formation and maturation of a competent mitochondrial network. It has previously been shown that the organization of the mitochondrial network changes as cardiomyocytes mature, such that in less mature cells the mitochondria exhibit a variety of random, perinuclear, and transcellular arrangements, whereas, in more mature cardiomyocytes, the mitochondria become intercalated in between the myofibrils, forming functional energetic units that facilitate energy production and EC-coupling during contraction [24]. As a consequence, it has been suggested that the localization and structure (which subsequently affect the function) of the mitochondria in cardiomyocytes are good indicators of the stage of differentiation or maturity of cells [50]. While the metabolic mechanisms that drive cardiac development are still largely unknown, it has been previously shown that a combination of *β*-adrenergic stimulation and fatty acid supplementation to mimic the postnatal development process leads to increased mitochondrial energetics [23]. Chung et al. [24] nicely demonstrated the intercalation of the mitochondria with the myofibrils using line scan analysis. We have now taken this one step further and quantified the level of intercalation using Pearson correlation coefficient, which is frequently used in biometrics to provide a measure of colocalization of two fluorescent signals [51] but in this case was used to indicate the amount of separation (or exclusion) of two signals. The scatter graphs shown in Figure 4 illustrate the level of intercalation along line scans from 4 distinct regions in each of 20 cells for each of the untreated controls and the various isoproterenol and oleic acid treatments. Our data clearly show that, even within one cell, the level of localization of mitochondria and myofibrils might vary considerably in different regions. This is especially so in the untreated cells and in those treated with isoproterenol alone (Figures 4(c) and 4(d)). When cells were treated with oleic acid (at either 100 *μ*M or 200 *μ*M), however, a higher level of intercalation was observed, as shown by the higher number of lines exhibiting a negative Pearson correlation value than a positive value (Figures 4(e) and 4(f)). Cells treated with a combination of isoproterenol and oleic acid (again at either 100 *μ*M or 200 *μ*M) showed an even greater level of mitochondria intercalation with over twice the number of line scans having a negative Pearson correlation than positive (Figures 4(g) and 4(h)).

## 5. Conclusions

In conclusion, we used four different morphometric methods to quantify the level of maturation of hESC-vCMs that were either untreated or else treated with the metabolic stimulants, isoproterenol and oleic acid, either alone or in combination. Using these quantification methods, we showed that these metabolic stimulants had an effect on some aspects of a cell's morphology but not on others. For example, isoproterenol had an effect on cell shape but did not seem to affect the cell area, the organization of the myofibrils, and (therefore perhaps somewhat unsurprisingly) the intercalation of mitochondria with the myofibrils. On the other hand, oleic acid (at both concentrations used) had a clear effect on cell shape, the orientation of the myofibrils, and the intercalation of the mitochondria with the myofibrils. When isoproterenol and oleic acid were used together, they had an obviously synergistic effect on cell shape and mitochondrial intercalation but perhaps less of an effect on the orientation of the myofibrils. None of these treatments, however, appeared to affect the cell area.

We suggest that these quantitative methods might provide simple and effective tools by which morphological maturation data can be compared both within one type of cell and between different cell types. Because of the wide variation in the data obtained for individual cells within the population, it is particularly important to show the distribution of the data in order to conduct accurate data analysis. In this way, changes in the overall trend of the data can be detected more clearly than if means and standard deviations (or standard errors) are presented. We suggest that these simple morphometric techniques might be adopted by other researchers in the field to more accurately describe the state of morphological maturation of ESC-CMs and iPSC-CMs in addition to the qualitative data that are already presented.
